# IL-17F depletion accelerates chitosan conduit guided peripheral nerve regeneration

**DOI:** 10.1186/s40478-021-01227-1

**Published:** 2021-07-17

**Authors:** Feixiang Chen, Weihuang Liu, Qiang Zhang, Ping Wu, Ao Xiao, Yanan Zhao, Ying Zhou, Qiaona Wang, Yun Chen, Zan Tong

**Affiliations:** 1 Department of Physiology and Medical Research Center for Structural Biology, School of Basic Medical Sciences, Wuhan University, Wuhan, China; 2 Department of Biomedical Engineering, Hubei Province Key Laboratory of Allergy and Immune Related Diseases, School of Basic Medical Sciences, Wuhan University, Wuhan, China; 3 Hangzhou Singclean Medical Products Co., Ltd., Hangzhou, China

**Keywords:** IL-17F, Macrophage polarization, Peripheral nerve regeneration, Nerve guidance conduit, Immune microenvironment

## Abstract

Peripheral nerve injury is a serious health problem and repairing long nerve deficits remains a clinical challenge nowadays. Nerve guidance conduit (NGC) serves as the most promising alternative therapy strategy to autografts but its repairing efficiency needs improvement. In this study, we investigated whether modulating the immune microenvironment by Interleukin-17F (IL-17F) could promote NGC mediated peripheral nerve repair. Chitosan conduits were used to bridge sciatic nerve defect in IL-17F knockout mice and wild-type mice with autografts as controls. Our data revealed that IL-17F knockout mice had improved functional recovery and axonal regeneration of sciatic nerve bridged by chitosan conduits comparing to the wild-type mice. Notably, IL-17F knockout mice had enhanced anti-inflammatory macrophages in the NGC repairing microenvironment. In vitro data revealed that IL-17F knockout peritoneal and bone marrow derived macrophages had increased anti-inflammatory markers after treatment with the extracts from chitosan conduits, while higher pro-inflammatory markers were detected in the Raw264.7 macrophage cell line, wild-type peritoneal and bone marrow derived macrophages after the same treatment. The biased anti-inflammatory phenotype of macrophages by IL-17F knockout probably contributed to the improved chitosan conduit guided sciatic nerve regeneration. Additionally, IL-17F could enhance pro-inflammatory factors production in Raw264.7 cells and wild-type peritoneal macrophages. Altogether, IL-17F may partially mediate chitosan conduit induced pro-inflammatory polarization of macrophages during nerve repair. These results not only revealed a role of IL-17F in macrophage function, but also provided a unique and promising target, IL-17F, to modulate the microenvironment and enhance the peripheral nerve regeneration.

**Supplementary Information:**

The online version contains supplementary material available at 10.1186/s40478-021-01227-1.

## Introduction

Peripheral nerve injury (PNI) is a serious health problem usually caused by trauma and medical disorders, and often leads to flexible neuropathies or permanent disability [1]. Repairing long nerve deficits remains a clinical challenge nowadays. The most widely used technique is the autograft, which is considered the golden standard for nerve repair [23]. However, the availability of donor nerves and loss of donor nerve function limit the application of the autograft strategy [19]. Therefore, artificial nerve guidance conduits (NGCs) are developed to bridge long nerve gaps [36]. Various strategies are applied to enhance the nerve repair effects including optimizing the biomaterial of conduits, introducing topographical cues, local delivery of neurotrophic factors or/and seed cells [30, 31, 38, 42, 48]. Special attentions have been paid to immune modulatory strategies especially macrophage-based methods, since macrophages play essential roles during nerve degeneration and regeneration after PNI [9, 25]. Ablation of macrophages is detrimental to axonal debris clearance and regenerative microenvironment reconstruction after nerve injury [2, 4, 22].

Macrophages demonstrate different phenotypes that are highly dependent on the changing microenvironment after nerve injury [34]. Based on their properties, macrophages could be generally categorized into pro-inflammatory and anti-inflammatory macrophages [26, 27]. Pro-inflammatory macrophages exhibit pro-inflammatory and distal neurodegenerative functions, while anti-inflammatory macrophages associate with anti-inflammatory and proximal axonal pro-healing effects [7, 8, 47]. Although both pro-inflammatory and anti-inflammatory are required during nerve repair, anti-inflammatory macrophages favored strategies seem to provide better regenerative effects [6, 14]. Local delivery of Interleukin-4 (IL-4) within polymeric nerve guidance conduits could enhance Schwann cells infiltration and substantially promote nerve regeneration in a critically-sized (15 mm) rat sciatic nerve gap repair [25]. The effects of IL-4 (promoting anti-inflammatory macrophages polarization) on axonal growth during conduit guided nerve repair were better than those of Interferon gamma (promoting pro-inflammatory macrophages polarization) [25]. It is reported that NGC with aligned nanofibers facilitated nerve regeneration with enhanced anti-inflammatory macrophages, while NGC with random nanofibers guided much weaker nerve regeneration with more pro-inflammatory macrophages during long gap peripheral nerve bridging [14]. Therefore, identifying a means of balancing pro-inflammatory and anti-inflammatory macrophages in the microenvironment is critical for developing efficient strategies to repair PNI.

Interleukin-17F (IL-17F) is an inflammatory cytokine produced not only by activated T cells, but also by activated monocytes [15, 32]. IL-17F belongs to the IL-17 family and shares the highest amino acid sequence homology (about 50%) with IL-17A among the six members [16]. IL-17F was reported to enhance granulopoiesis and induce the production of many pro-inflammatory cytokines and chemokine [5, 41]. IL-17A and IL-17F stimulation induced anti-inflammatory phenotype in human macrophages but not in murine macrophages [10]. Despite this knowledge, the role of IL-17F in macrophages, especially under the nerve repair condition has been poorly studied.

In this study, we investigated whether IL-17F depletion could facilitate conduit guided sciatic nerve recovery and the possible mechanism regarding pro-inflammatory and anti-inflammatory macrophage polarization. Sciatic nerve defect was generated in *Il17f* ^−/−^ mice and wild-type mice. Then chitosan conduits [46] were applied to bridge the nerve gaps with autografts as control. The functional recovery and axonal regeneration of sciatic nerve were compared between the knockout and wild-type mice. Meanwhile, the phenotypes of macrophages were assessed both in vivo and in vitro.

## Materials and methods

### Preparation of the chitosan materials

The chitosan conduits were prepared as previous work [46]. In brief, 1% chitosan solution was prepared for electrodeposition and chitosan was deposited on the cathode during the electrolysis process. Chitosan conduits with single channel and 0.5 mm inner diameter were generated by using stainless-steel needle with 0.5 mm diameter as the cathode. Chitosan films were generated by changing the cathode into stainless-steel plate. Conduits were dried, weighed, then steam-sterilized before extraction and the extraction ratio was 0.2 g conduits per 1 mL medium. Extracted medium was prepared by shaking the conduits in cell culture medium (10%FBS, DMEM) at 15 rpm 37 °C for 72 h, and then the supernatants were collected after centrifugation at 1000 rpm for 10 min. Cell culture medium without conduits applied to the same procedure was used as control.

### Animals

8 ~ 10-week-old male *Il17f* ^−/−^ (KO) [11, 41] and wild-type (WT) C57BL/6 mice were used in this study. Mice were kept in pathogen-free facilities on a 12-h light–dark cycle. All protocols and procedures in this study were approved by the Ethics Committee of Wuhan University (Permit Number: AF047).

### Surgical procedure

**Fig. 1:**
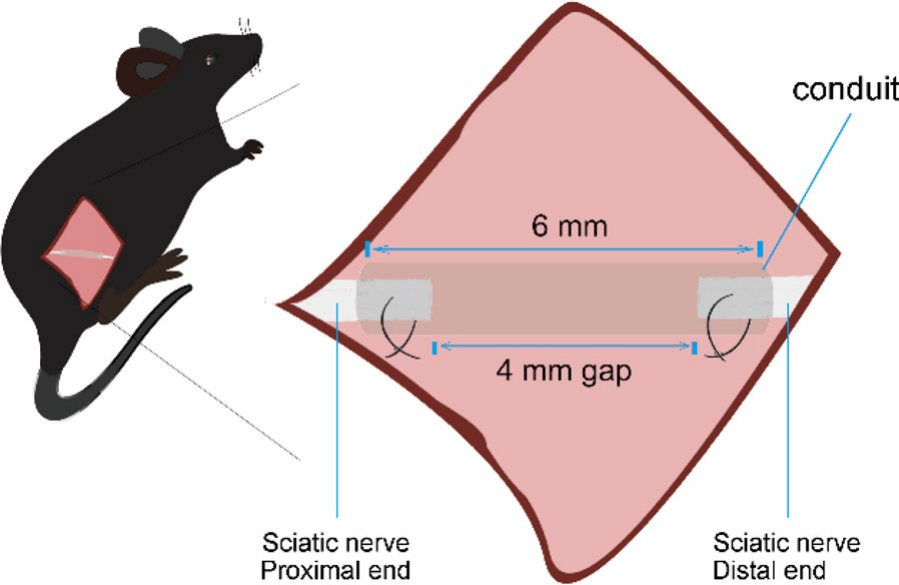
Chitosan conduit mediated sciatic nerve transection repair

Ten KO mice and ten WT mice were used for chitosan conduit mediated sciatic nerve repair. Ten KO mice and ten WT mice were used for autograft. The mice were anesthetized intraperitoneally during all surgical procedures. The right-side sciatic nerve was exposed after the skin incision and muscle separation. In chitosan conduit groups, the sciatic nerve was transected and a 6 mm-long conduit was sutured between proximal and distal nerve segments creating a 4 mm gap (Fig. 1). In autograft groups, the transected sciatic nerve was reversely sutured between proximal and distal nerve ends.

### Sciatic function index evaluation

Walking track analysis were performed at 12 and 20 weeks after surgery. The hind paws of mice were dipped in red ink. Then mice were allowed to freely walk multiple times and leave prints on white papers. The sciatic function index (SFI) was calculated as previous reported [45].

### Electrophysiological detection

Electrophysiology experiment was performed at 20 weeks after surgery. The sciatic nerves of the operated side were re-exposed under anesthesia. The electromyography was recorded by biological signal collecting system (RM6240, China). The 10 mV 1 kHz electrical stimuli were applied to the nerve trunk at the proximal end. While compound muscle action potentials (CMAPs) were recorded on the gastrocnemius muscle. The amplitude and latency of CMAPs in each group were used to assess the sciatic nerve functional recovery.

### Histological assessment of regenerated nerve

Immediately after the electrophysiological study, the mice were sacrificed and the regenerated nerves were collected for toluidine blue staining, transmission electron microscopy (TEM) and immunohistochemical staining as previous report [44]. Toluidine blue staining sections were randomly chosen for analysis of diameter and density of myelinated nerve fibers and TEM sections were randomly chosen for analysis of the area of the myelinated axons and myelin sheath thickness by Image-Pro Plus software (Media Cybernetics, Inc., USA).

### Morphometric analysis of gastrocnemius muscles

Immediately after collection of nerves, the gastrocnemius muscles of both operated and normal sides were dissected, weighed and photographed. The muscle weight recovery rate was calculated by the percentage of operated side muscle weight to normal side muscle weight. The muscle samples were sectioned for Masson’s trichrome staining. The muscle fibers area (Red) and the collagen fiber area (Blue) on the sections were analysed by Image-Pro plus software (Media Cybernetics, Inc., USA).

### Cell culture and analysis

Raw264.7 cell growth were monitored by Real-Time Cell Analyzer (RTCA, xCELLigence, Roche). Briefly, 3 * 10^3^ cells were seeded in E-plates 96 with different culture media. The cell growth index in each well was recorded by the software supplied with the instrument. Cell cycle were analysed by flow cytometry (BD AriaIII, USA) and the results were analysed using Modifit LT software (Verity Software House, USA). Immunofluorescence staining was performed as previously described [39]. The cells were incubated with rabbit anti-Ki67 (Abcam, 0.8 mg/ml, 1:100 dilution), Nos2 (Abcam, 4 mg/ml, 1:100 dilution), or Arg1 (Abcam, 1 mg/ml, 1:100 dilution) antibody at 4 °C overnight. After washed by PBS, the cells were incubated with FITC conjugated goat anti-rabbit IgG (Boster, 1 mg/ml, 1:200 dilution) at 37 °C for 1 h. The nuclei were stained by DAPI and observed under confocal laser scanning microscope (Leica-LCS-SP8-STED, Leica, Germany). Macrophages were treated with 0, 12.5, 25, 50, 100, 200 ng/ml mouse IL-17F protein (BioLegend, USA) for 24 h before collected for qPCR analysis.

### Primary macrophages isolation and culture

Peritoneal macrophages (PeM) and Bone marrow derived macrophages (BMDM) were isolated as our previously described [21]. Briefly, 5 ml medium (10%FBS, DMEM) was injected into the peritoneal cavity of each mice. Fluid from peritoneal cavity was collected and cells were washed, suspended and counted. 5 ml medium (10%FBS, DMEM) was injected into the femoral and tibial bone marrow of each mice. Cells were collected after red cell lysis using ACK lysing buffer (Gibco, USA), and further washed, suspended and counted. 2 * 10^6^ cells were allowed to adhere to each 24 well culture plate 2 h at 37 °C. Non-adherent cells were removed by gently washing three times with warm PBS. Then, the macrophages were cultured in the 10%FBS, DMEM with different treatments for 24 h.

### ELISA

Raw264.7 cells were cultured with conduit extracted medium, control medium or fresh medium for 24 h and the supernatants were collected after centrifugation. Mouse IL-1b, IL-6, IL-10 or Vegf concentrations in the supernatants were determined using ELISA kits as recommended by the assay manufacturer (Bio-swamp, China).

### Quantitative RT-PCR

Macrophages and regenerated nerves were collected for RNA expression analysis. RNA was extracted using Trizol method and reverse transcripted to cDNA using RT Kit (TOYOBO CO., LTD, Osaka, Japan). Quantitative PCR (qPCR) analysis were performed on Bio-Rad CFX Connect System using the SYBR Green PCR Master Mix (Takara). The relative mRNA level of each gene was normalized against *β-actin* and *Rn18S*. The primer sequences for qPCR amplification were listed in the Additional file 1: Table S1.

### Statistical analysis

All values were expressed as mean ± standard derivation (Mean ± SD). Statistical significances were determined by Student’s t-test analysis for comparing two groups and one-way analysis of variance (ANOVA) for multiple groups with GraphPad Prism 8 software (GraphPad Software, Inc., CA, USA). *P* < 0.05 was considered statistically significant.

## Results

### IL-17F knockout improved functional recovery of sciatic nerve bridged by chitosan conduits

**Fig. 2:**
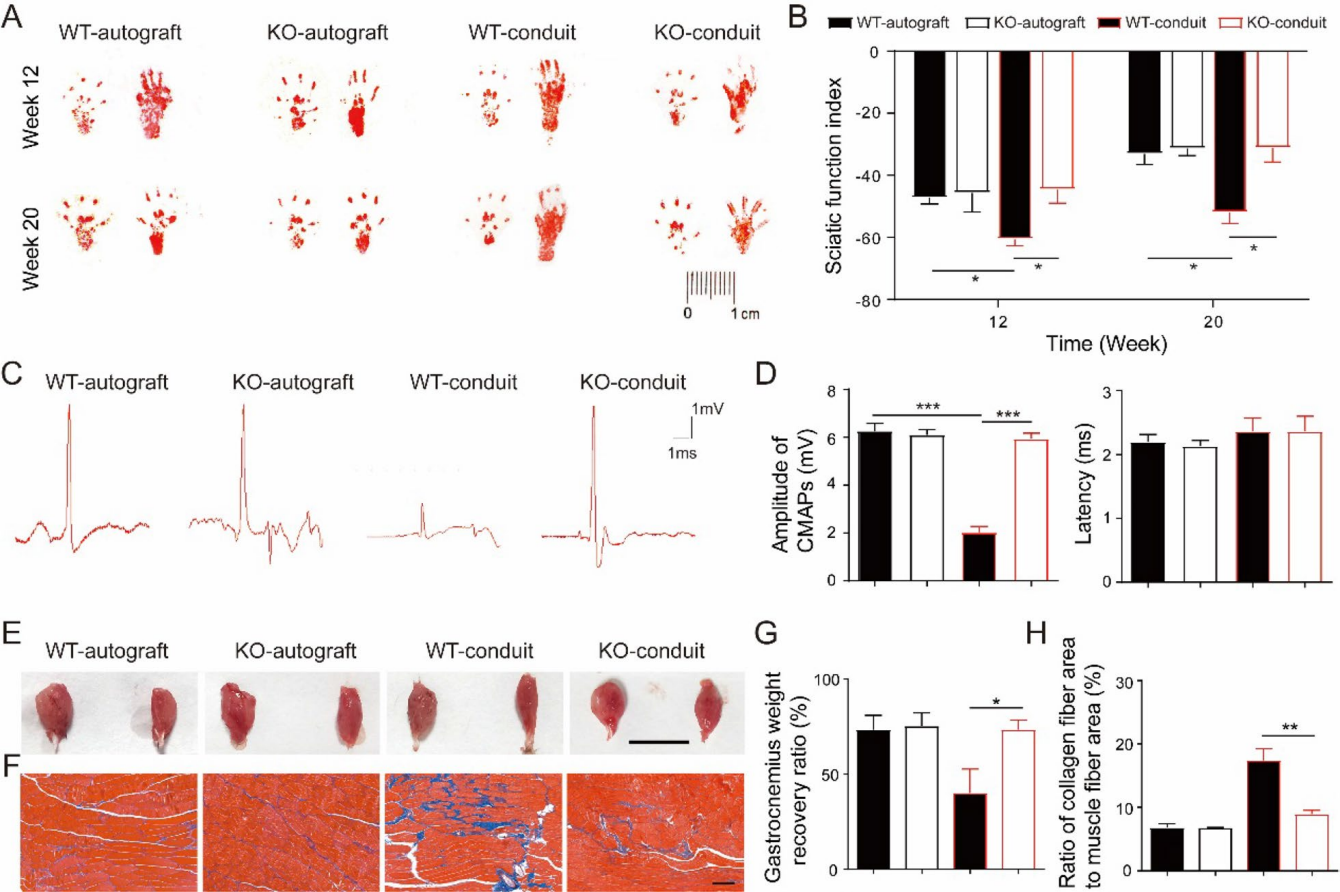
KO mice had improved functional recovery of sciatic nerve comparing to WT mice. **a** Representative image of walking track. **b** SFI analysis. N = 10. **c** Representative CMAPs recorded on the regenerated nerve. **d** Analysis of peak amplitude and latency of CMAPs. N = 3. **e** Images of gastrocnemius muscle from both normal (left) and operative (right) sides. Bar = 1 cm. **f** Masson’s trichrome staining of gastrocnemius muscle sections. Bar = 100 μm. **g** The gastrocnemius weight recovery ratio. N = 10. **h** The ratio of collagen fiber area to muscle fiber area. N = 3. All values are expressed as Mean ± SD. *P < 0.05; **P < 0.01; ***P < 0.001

Chitosan conduit were applied to bridge the sciatic nerve gaps in the KO and WT mice, while autograft groups were used as control. Walking track were recorded at 12 weeks and 20 weeks after surgery (Fig. 2a). Sciatic function index (SFI) was calculated by analysis the walking track (Fig. 2b). There was no significant difference of SFI values between the KO-autograft and WT-autograft mice. Lower SFI values in WT-conduit mice were observed comparing to those in WT-autograft mice at both 12 and 20 weeks after surgery. However, the SFI values in KO-conduit mice were significantly higher than those in WT-conduit mice, and similar to autograft groups. These results suggested that the KO mice had improved motor functional recovery of sciatic nerve bridged by chitosan conduits comparing to WT mice.

Twenty weeks after surgery, electrophysiological studies were performed and the representative electrophysiological records were shown in Fig. 2c. The peak amplitudes of CMAPs in KO-conduit mice were significantly higher than those in WT-conduit mice and similar to autograft groups, while latencies of CMAPs in all four groups showed no significant difference (Fig. 2d). Immediately after electrophysiological studies, the gastrocnemius muscles on both normal and operative sides were taken out, weighed and photographed (Fig. 2e). The muscle sizes on the operation side were smaller than those on the normal side in all four groups. The muscle weight recovery ratio of the gastrocnemius in the KO-conduit mice was higher than that in the WT-conduit mice and similar to autograft groups (Fig. 2g). Further Masson’s trichrome staining exhibited that gastrocnemius muscle fibers were neatly arranged and uniform in size with little collagen fibers in both WT and KO autograft groups (Fig. 2f). However, large area of collagen fibers was observed in WT-conduit mice, while a few collagen fibers in KO-conduit mice. The ratio of collagen fiber area to muscle fiber area in KO-conduit mice was lower than that in WT-conduit mice and similar to autograft groups (Fig. 2h). These results suggested that KO mice had greatly improved neuromuscular reinnervation ability comparing to WT mice during chitosan conduit guided sciatic nerve repair.

### IL-17F knockout promoted axonal regeneration of sciatic nerve bridged by chitosan conduits

**Fig. 3:**
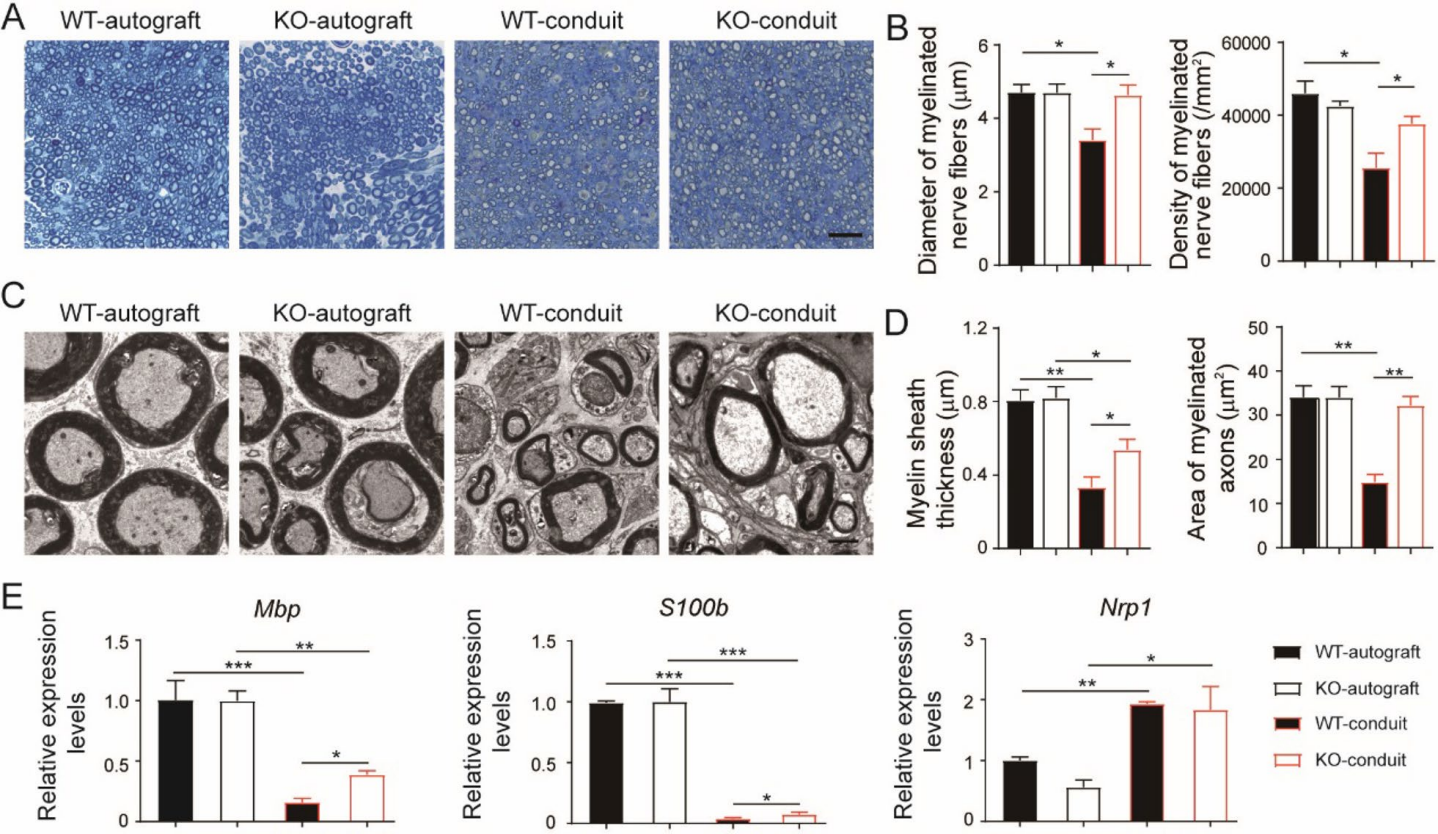
KO mice had improved axonal regeneration of sciatic nerve comparing to WT mice. **a** Toluidine blue staining of regenerated nerves. Bar = 20 μm. **b** Analysis of the myelinated nerve fibers based on toluidine blue staining. N = 3. **c** TEM images of regenerated nerves. Bar = 2 μm. **d** Analysis of the myelinated nerve fibers based on TEM images. N = 3. **e** Quantitative PCR analysis of regenerated nerves. N = 3. All values are expressed as Mean ± SD. *P < 0.05; **P < 0.01; ***P < 0.001

To assess the myelination during the sciatic nerve regeneration, toluidine blue staining and TEM analysis of the regenerated nerves were performed at 20 weeks after surgery. Better structures of the nerves were observed in autograft groups comparing to conduit groups (Fig. 3a, c). To gain a comprehensive understanding of the nerve regeneration, four parameters were calculated: diameter and density of myelinated nerve fibers, myelin sheath thickness, and area of myelinated axons. The diameters and the density of the myelinated nerve fibers in KO-conduit mice were bigger than those in WT-conduit mice and similar to autograft groups (Fig. 3b). The area of the myelinated axons in KO-conduit mice was also bigger than those in WT-conduit mice and similar to autograft groups (Fig. 3d). However, the myelin sheath thickness in the conduit groups were smaller than those in the autograft groups both in KO and WT mice. Meanwhile, the myelin sheath thickness in KO-conduit mice were bigger than those in WT-conduit mice.

To obtain a quantitative understanding of the nerve regeneration, qPCR analysis of the regenerated nerves were performed (Fig. 3e). The expression levels of two critical myelination related factors, myelin basic protein (*Mbp*) and *S100b*, were dramatically lower in regenerated nerves from conduit groups comparing to autograft groups. At the same time, KO-conduit mice had higher expression levels of *Mbp* and *S100b* comparing to WT-conduit mice. On the other hand, the expression levels of nerve injury related factor, neuropilin 1 (*Nrp1*), were significantly increased in regenerated nerves from conduit groups comparing to autograft groups and there was no difference between KO and WT mice.

All these results indicated that the IL-17F knockout was more favorable for axon outgrowth and remyelination than wild-type control during chitosan conduit guided sciatic nerve regeneration, despite the conduit guided groups showed worse axonal regeneration of sciatic nerve comparing to autograft groups.

### IL-17F knockout enhanced anti-inflammatory macrophages in chitosan conduit guided sciatic nerve repairing microenvironment

**Fig. 4:**
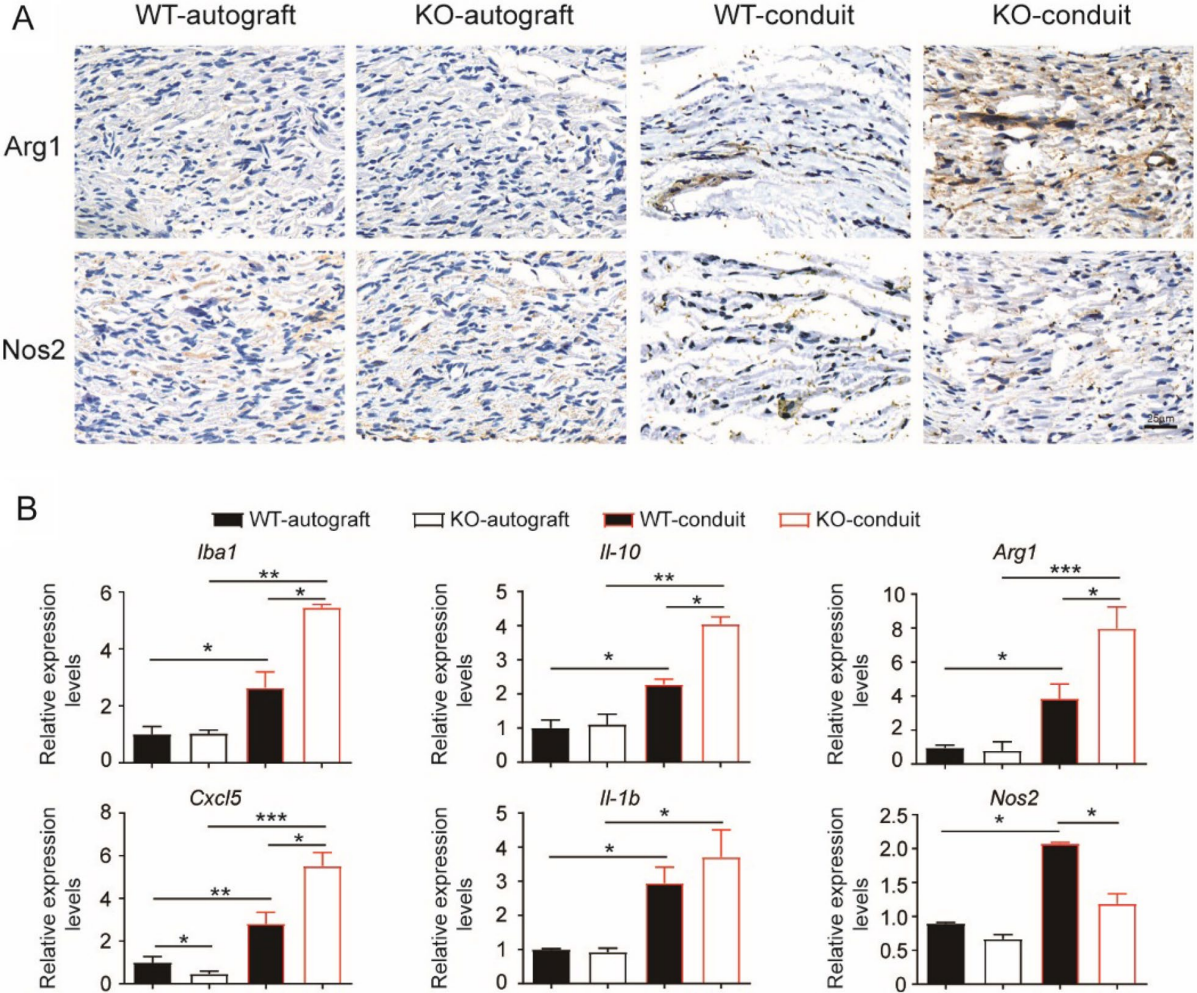
KO mice had enhanced anti-inflammatory macrophages in chitosan conduit guided sciatic nerve repairing microenvironment. **a** Immunohistochemical staining of Arg1 and Nos2 in the regenerated nerves. Bar = 25 μm. N = 3. **b** Quantitative PCR analysis of the regenerated nerves. N = 3. All values are expressed as Mean ± SD. *P < 0.05; **P < 0.01; ***P < 0.001

The phenotypes of macrophages in the regenerated nerves were investigated both in KO and WT mice at 20 weeks after surgery. Hardly any macrophages could be detected by immuno-staining in the regenerated nerves of autograft groups, while macrophages were detectable in conduit groups (Fig. 4a). Macrophages in the regenerated nerves from KO-conduit mice highly expressed the anti-inflammatory marker, Arg1. In contrast, macrophages in the regenerated nerves from WT-conduit mice expressed both Arg1 and the pro-inflammatory marker, Nos2.

qPCR analysis of the regenerated nerves revealed that there was no difference of *Iba-1*, *Il-10*, *Arg1*, *Il-1b* and *Nos2* expression levels between KO and WT mice in the autograft groups (Fig. 4b). Conduit groups had increased mRNA levels of *Iba-1*, *Il-10*, *Arg1*, *Il-1b* and *Nos2* compared with autograft groups suggesting elevated macrophages response in regenerated nerves induced by chitosan conduit. In the conduit groups, regenerated nerves from KO mice had higher expression levels of *Iba-1*, *Il-10*, and *Arg1* and lower expression levels of *Nos2* comparing to those from WT mice (Fig. 4b). These results suggested that chitosan conduits induced inflammation and the polarization of both pro-inflammatory and anti-inflammatory macrophages, and IL-17F knockout biased the anti-inflammatory polarization in chitosan conduit guided nerve repairing microenvironment.

On the other hand, regenerated nerves from KO mice had decreased expression levels of *Cxcl5*, a neutrophil-recruiting chemokine. However, regenerated nerves from KO mice had higher expression levels of *Cxcl5* comparing to those from WT mice in the conduit groups (Fig. 4b). These results suggested that IL-17F may also play a role in neutrophil modulation in chitosan conduit guided nerve repairing microenvironment.

### Chitosan conduits inhibited the growth and induced the polarization to pro-inflammatory phenotype of Raw264.7 cells in vitro

**Fig. 5:**
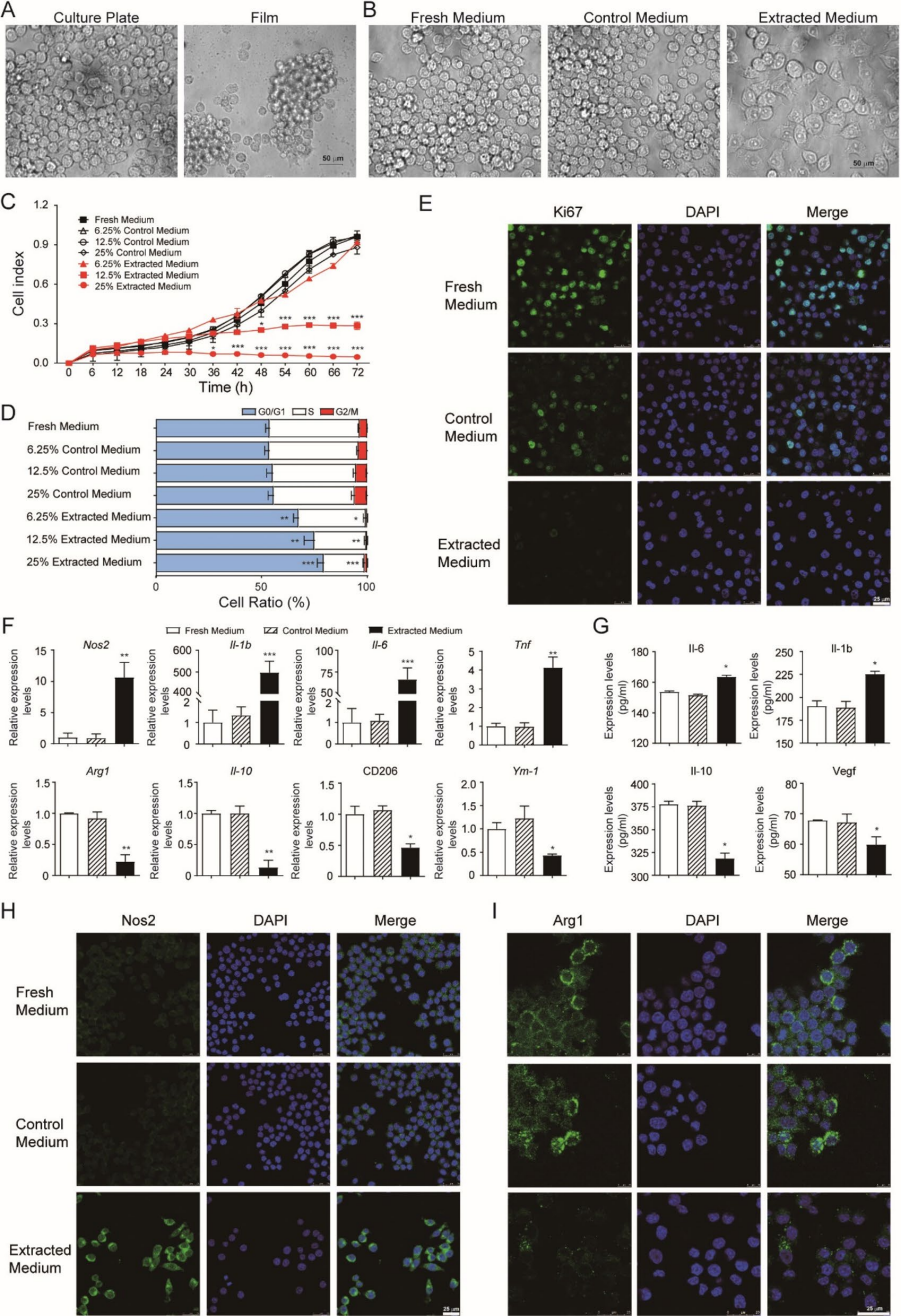
Chitosan conduits affected the growth and polarization of Raw264.7 macrophage cells. **a** Light microscope images of cells cultured on the plate and chitosan film. **b** Light microscope images of cells cultured in 25% different media for 24 h. **c** RTCA analysis of cells cultured in different media. N = 3. **d** Cell cycle analysis by flow cytometry of cells cultured in different media for 24 h. N = 3. **e** Immunofluorescence images of cells cultured in 25% different media for 24 h. **f** Quantitative PCR analysis of cells cultured in 25% different media for 24 h. N = 5. **g** ELISA analysis of the supernatants from cells cultured in 25% different media for 24 h. N = 3. **h**, **i** Immunofluorescence images of cells cultured in 25% different media for 24 h. All values are expressed as Mean ± SD. *P < 0.05; **P < 0.01; ***P < 0.001; comparing to control medium with the same concentration

The effects of chitosan conduits on macrophages in vitro were further explored. The mouse macrophage Raw264.7 cells could hardly attach to the chitosan film, which had only shape difference with chitosan conduits (Fig. 5a). Therefore, the extracted medium form chitosan conduits was prepared and used for cell culture. After 24 h culture, the Raw264.7 cells got a differentiated phenotype in the extracted medium, while cells remained round and bright in the fresh medium or control medium (Fig. 5b). At the same time, the Raw264.7 cell growth was significantly inhibited in the 12.5% extracted medium, and there was hardly any cell growth in the 25% extracted medium (Fig. 5c). Further cell cycle analysis revealed that the ratios of cells in G0/G1 phase were increased, while the ratios of cells in S phase were decreased corresponding to the increased percentage of extracted medium (Fig. 5d). Immunofluorescence staining demonstrated that only 5.0 ± 0.4% Ki67 positive cells could be detected in cells cultured in the extracted medium, while 48.7 ± 2.9% Ki67 positive cells were observed in control medium and 82.3 ± 1.7% Ki67 positive cells in fresh medium (Fig. 5e). These results indicated that the extracted medium from chitosan conduit could efficiently suppress Raw264.7 cell growth by both cell cycle arrest and proliferation inhibition.

The effects of chitosan extracts on the polarization of macrophages in vitro were further investigated. The Raw264.7 cells were analysed after cultured in 25% chitosan conduit extracted medium or 25% control medium or fresh medium for 24 h. The mRNA levels of pro-inflammatory markers, *Nos2*, *Il-1b*, *Il-6* and *Tnf* were dramatically increased, while the levels of anti-inflammatory markers, *Arg1*, *Il-10*, CD206 and *Ym-1* were significantly decreased in the cells after the extracts treatment (Fig. 5f). ELISA analysis of the supernatant confirmed increased protein levels of Il-6 and Il-1b and decreased protein levels of Il-10 and Vegf in the cells after the extracts treatment (Fig. 5g). Immunofluorescence staining demonstrated that plenty of Nos2 positive cells were observed in cells cultured in the extracted medium, while rare Nos2 positive cells could be detected in cells cultured in fresh medium or control medium (Fig. 5h). In contrast, less Arg1 positive signals could be detected in cells cultured in the extracted medium (Fig. 5i). These results suggested that the extracted medium from chitosan conduit induced the polarization of Raw264.7 cells to pro-inflammatory phenotype.

### IL-17F knockout increased the anti-inflammatory markers in peritoneal macrophages and bone marrow derived macrophages after treatment with the extracts from chitosan conduits

**Fig. 6:**
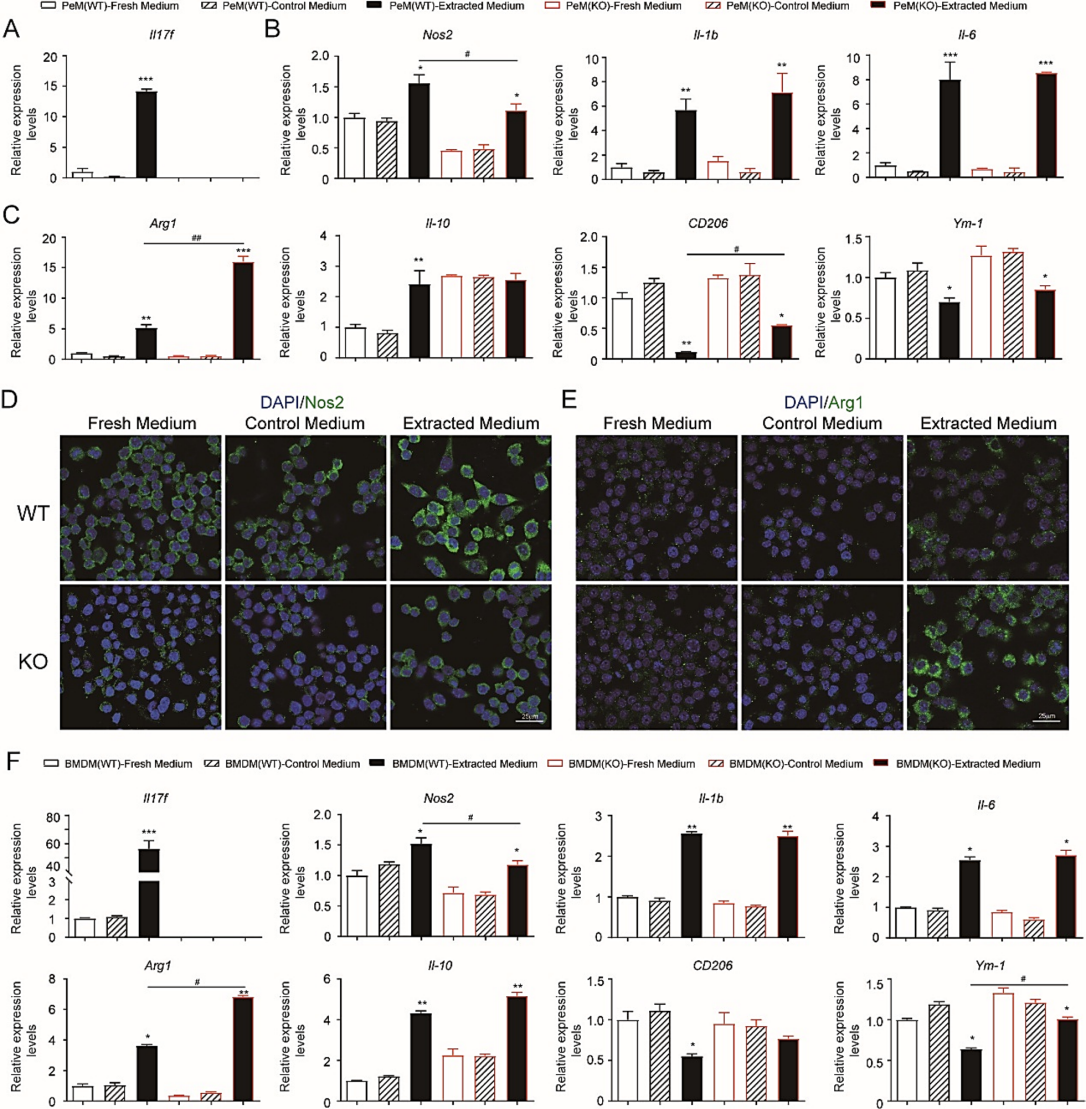
Peritoneal macrophages (PeM) and Bone marrow derived macrophages (BMDM) from KO and WT mice polarized differently after chitosan conduits extracts treatment. **a**, **c** Quantitative PCR analysis of PeM cells treated with 25% different media for 24 h. N = 5. **d**, **e** Immunofluorescence images of cells treated with 25% different media for 24 h. **f** Quantitative PCR analysis of BMDM cells treated with 25% different media for 24 h. N = 3. All values are expressed as Mean ± SD. *P < 0.05; **P < 0.01; ***P < 0.001; comparing to control medium. #*P* < 0.05; ##*P* < 0.01

In order to clarify the effects of IL-17F knockout on macrophages, KO and WT peritoneal macrophages and bone marrow derived macrophages were examined and compared. WT peritoneal macrophages had dramatically increased expression levels of *Il17f* after the extracts treatment (Fig. 6a). The mRNA levels of pro-inflammatory markers, *Nos2*, *Il-1b*, and *Il-6* were significantly increased in both KO and WT peritoneal macrophages after the extracts treatment (Fig. 6b). However, the levels of *Nos2* were significantly lower in KO peritoneal macrophages comparing to WT after the extracts treatment. At the same time, the mRNA levels of anti-inflammatory markers, *Arg1* were also increased in both KO and WT peritoneal macrophages after the extracts treatment (Fig. 6c). However, the levels of *Arg1* increased more striking in KO peritoneal macrophages and were significantly higher than WT peritoneal macrophages after the extracts treatment. When IL-17F was knockout, peritoneal macrophages had higher *Il-10* mRNA levels than WT controls. After the extracts treatment, WT peritoneal macrophages had increased expression level of *Il-10*, while the KO peritoneal macrophages had unaltered expression level of *Il-10* (Fig. 6c). Therefore, there was no significant difference of *Il-10* levels between KO and WT peritoneal macrophages after the extracts treatment. In addition, KO peritoneal macrophages had higher levels of CD206 and *Ym-1* than WT peritoneal macrophages after the extracts treatment.

Immunofluorescence staining demonstrated that enhanced Nos2 positive signals were observed in both KO and WT peritoneal macrophages after the extracts treatment, while relatively weaker Nos2 signals could be detected in KO peritoneal macrophages comparing to WT peritoneal macrophages (Fig. 6d). At the same time, enhanced Arg1 positive signals were also observed in both the KO and WT peritoneal macrophages after the extracts treatment. However, relatively stronger Arg1 signaling could be detected in KO peritoneal macrophages comparing to WT peritoneal macrophages (Fig. 6e).

Bone marrow derived macrophages (BMDM) from KO and WT mice were used to confirm the changes in peritoneal macrophages (Fig. 6f). WT BMDM had dramatically increased expression levels of *Il17f* after the extracts treatment. Similar changes of pro-inflammatory markers, *Nos2*, *Il-1b*, and *Il-6* were found in KO and WT BMDM. For anti-inflammatory markers, the higher levels of *Arg1* could also be observed in KO BMDM than WT BMDM after the extracts treatment. Moreover, the levels of *Ym-1* decreased and were higher in KO BMDM than in WT BMDM after the extracts treatment.

These in vitro findings corresponded well with the in vivo studies and provided strong evidence for the role of IL-17F in pro-inflammatory and anti-inflammatory macrophages switching during chitosan guided nerve regeneration.

### IL-17F enhanced pro-inflammatory factors production in macrophages

**Fig. 7:**
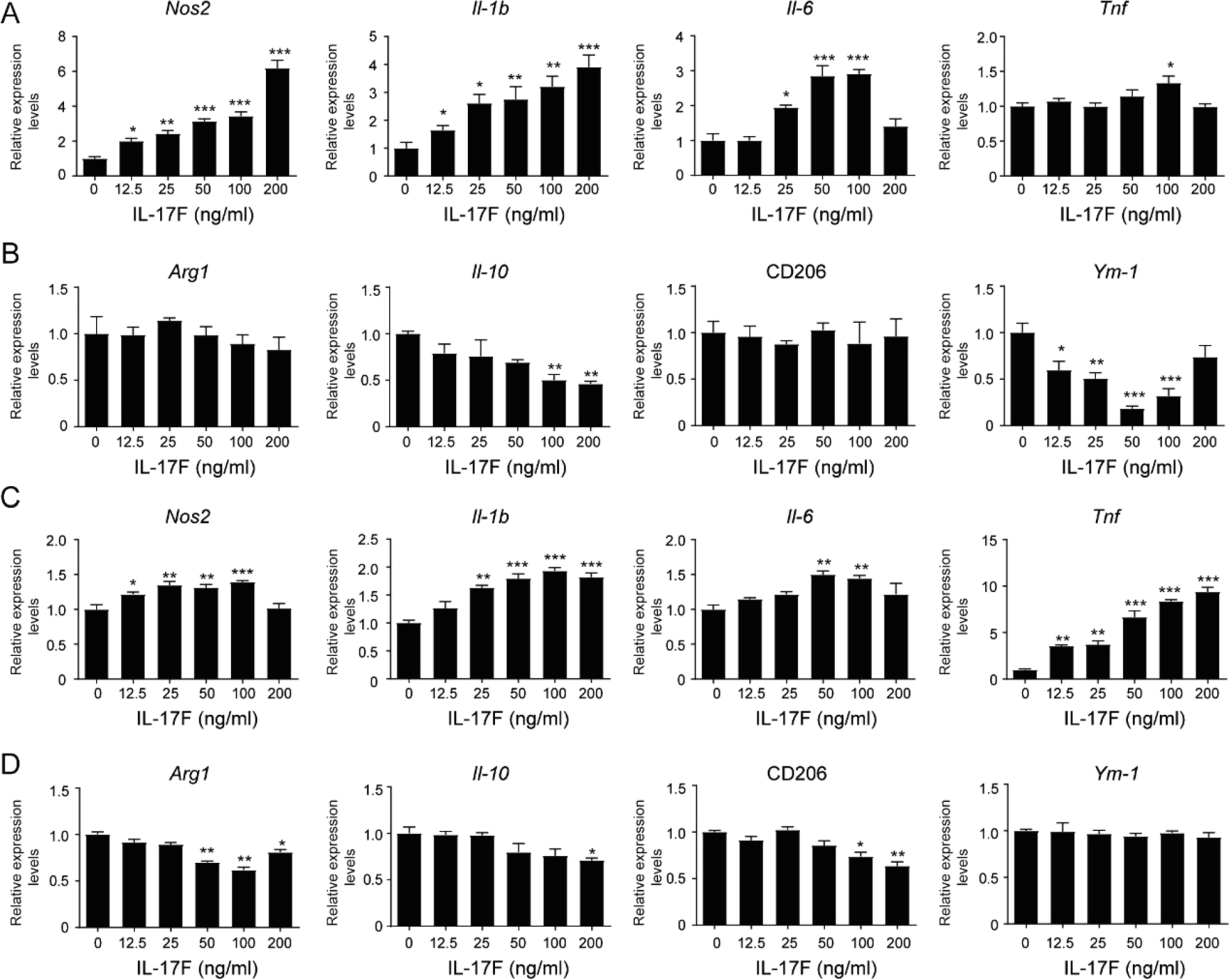
IL-17F induced pro-inflammatory factors production in Raw264.7 cells and peritoneal macrophages. **a**, **b** Quantitative PCR analysis of Raw264.7 cells treated with different concentration of IL-17F for 24 h. N = 3. **c**, **d** Quantitative PCR analysis of WT peritoneal macrophages treated with different concentration of IL-17F for 24 h. N = 3. All values are expressed as Mean ± SD. *P < 0.05; **P < 0.01; ***P < 0.001; comparing to 0 group

Direct effects of IL-17F on macrophages were further evaluated by treating Raw264.7 cells and WT peritoneal macrophages with different concentration of IL-17F. Raw264.7 cells showed IL-17F dose-dependent increase of *Nos2*, *Il-1b*, and *Il-6* expression levels (Fig. 7a). At the same time, Raw264.7 cells had decreased expression levels *of Il-10* after treatment with high concentration (100, 200 ng/ml) of IL-17F and decreased expression levels of *Ym-1* in IL-17F dosage dependent manner (Fig. 7b). Peritoneal macrophages showed IL-17F dose-dependent increase of *Nos2*, *Il-1b*, and *Tnf* expression levels (Fig. 7a). At the same time, peritoneal macrophages had decreased expression levels *of Arg1, Il-10* and CD206 after treatment with high concentration of IL-17F (Fig. 7b). Though there were some different responses to IL-17F stimulation between Raw264.7 cell line and peritoneal macrophages, overall data demonstrated that IL-17F promoted pro-inflammatory factors production in macrophages.

## Discussion

In this study, we found IL-17F depletion could promote chitosan conduit mediated sciatic nerve regeneration (Figs. 2, 3). Moreover, IL-17F depletion could reduce pro-inflammatory macrophages and enhance pro-regenerative macrophages in the microenvironment of chitosan conduit guided nerve repair (Fig. 4). In vitro data revealed that chitosan conduit could induce pro-inflammatory phenotypes of macrophages (Figs. 5, 6). IL-17F knockout macrophages exhibited more pro-regenerative phenotypes after chitosan conduit treatment (Fig. 6). Additionally, IL-17F could induce pro-inflammatory cytokines production in macrophages (Fig. 7). These in vivo and in vitro data, the knockout and supplement of IL-17F data all together support that IL-17F could modulate the immune microenvironment of chitosan conduit mediated nerve regeneration by altering chitosan induced pro-inflammatory polarization of macrophages.

Chitosan conduits were widely used in the clinic for guiding nerve regeneration in short gap injury of peripheral nerve. The reason for the failure of chitosan conduits application in long gap nerve injury repair has not been clarified. It is reported that chitosan had an in vitro stimulatory effect on nitric oxide (NO) production by macrophages [29] and chitosan oligosaccharide could stimulate macrophages through Toll like receptor 4 (TLR4) signaling pathway [43]. Since prolonged inflammation had adverse effects on myelin sheath and axon regeneration [17], chitosan conduit as exogenous agent stimulating prolonged pro-inflammatory reaction may contribute to its inefficiency in long gap nerve injury repair.

Chitosan conduit could stimulate peritoneal and bone marrow derived macrophage to secret IL-17F (Fig. 6), which is a relatively weak inflammatory cytokine comparing to IL-17A [12, 24]. IL-17F depletion had no effect on nerve regeneration in the autograft groups (Fig. 2, 3), which did not involve exogenous chitosan conduit stimulation. Moreover, IL-17F KO macrophages showed no significant difference in vitro with WT macrophages (Fig. 6). We chose the relatively longer time point to ensure almost fully recovery of sciatic nerve in the autograft groups and minimal differences between KO autograft and WT autograft groups. We will check more immune cell types at different time points after surgery in the future study. Since the exogenous NGC may easily stimulate the inflammatory response [33], combining NGC with IL-17F inhibitors may provide a useful strategy to improve NGC mediated peripheral nerve repair and regeneration.

In the present study, the roles of IL-17F in macrophages in vivo, macrophage cell line Raw264.7, and primary macrophages in vitro were examined. The different types of macrophages have different characteristics of cell growth. Macrophage cell line Raw264.7 showed fast proliferation capacity in vitro, which could be suppressed by chitosan conduit (Fig. 5). Meanwhile, peritoneal and bone marrow derived macrophages required stimulation to proliferate in vitro [37]. More macrophages in chitosan conduit guided nerves than autograft groups were detected (Fig. 4). These in vivo macrophages may probably recruited by the nerve injury signals, since the axonal regeneration were worse in the chitosan conduit groups (Fig. 3). More importantly, IL-17F altered the polarization of macrophages. IL-17F knockout biased the anti-inflammatory macrophages, while not totally blocked the pro-inflammatory macrophages after chitosan stimulation (Fig. 6). IL-17F supplement enhanced pro-inflammatory markers and reduced some anti-inflammatory markers in macrophages (Fig. 7). Since both pro-inflammatory and anti-inflammatory macrophages play pivotal roles during the peripheral nerve repair, completely depletion of pro-inflammatory macrophages function or knockout key inflammatory cytokine may not favor the nerve repair. For example, impaired functional recovery after nerve lesion were reported in the pro-inflammatory marker gene knockout mice, such as *Il-1β*^−/−^ and *Tnf*^−/−^, *inos*^−/−^ mice [18, 28]. Therefore, mixed pro-inflammatory and anti-inflammatory phenotypes with bias to anti-inflammatory of IL-17F knockout macrophages in the chitosan conduit induced microenvironment may have more advantages to improve nerve repair and regeneration.

Here, altered macrophage polarization by IL-17F knockout contributed to the improved chitosan conduit guided sciatic nerve regeneration. However, other changes caused by the global IL-17F knockout may also affect the sciatic nerve regeneration microenvironment including Th17 cells, neutrophils and endothelial cells. IL-17F is a pro-inflammatory effector together with IL-17A produced by Th17 cells [40]. It is reported that the Th17 cell frequency was increased in the regenerated nerve after mice sciatic nerve crush [3]. Moreover, T cells were found invaded into dorsal root ganglia after peripheral nerve lesions [13]. Therefore, IL-17F knockout may affect the Th17 cell function during the sciatic nerve regeneration. Besides, we found *Cxcl5*, a neutrophil-recruiting chemokine, had dramatically increased expression levels in the chitosan conduit guided nerve tissues and there were different changes of *Cxcl5* expression levels between KO and WT mice (Fig. 4). Since neutrophils were reported a role in myelin clearance at early stage after sciatic nerve transection [20], we will examine the role of IL-17F in neutrophils at initial stages of nerve regeneration in the future study. On the other hand, our previous study revealed IL-17F could inhibit angiogenesis [35], which is crucial in the peripheral nerve regeneration. Promoted angiogenesis by IL-17F knockout could be expected and may contribute to the enhanced repairing effects in this study. Altogether, comprehensive effects by IL-17F knockout may additively contribute to the improved chitosan conduit guided sciatic nerve regeneration. Further studies are needed, and our study represents a first step toward a better understanding of the roles of IL-17F in the sciatic nerve repairing immune micro-environment.

## Conclusions

In summary, our data revealed that IL-17F knockout improved chitosan conduit guided sciatic nerve regeneration with enhanced functional recovery and axonal myelination. IL-17F knockout modulated the chitosan conduit induced pro-inflammatory polarization of macrophages and favored anti-inflammatory phenotype of macrophages. IL-17F could enhance pro-inflammatory factors production in macrophages and may partially mediate chitosan conduit induced pro-inflammatory polarization of macrophages during nerve repair. Although further molecular mechanism and other immune cells need to be explored, our study provided a unique and promising target, IL-17F, to regulate the microenvironment and enhance the peripheral nerve regeneration.

## Supplementary Information

**Additional file 1**. **Table S1**; The primer sequences for qPCR amplification.

## Data Availability

The datasets used and/or analysed during the current study are available from the corresponding author on reasonable request.

## Abbreviations

IL-17F: Interleukin-17F
NGC: Nerve guidance conduit
PNI: Peripheral nerve injury
SFI: Sciatic function index
CMAPs: Compound muscle action potentials
TEM: Transmission electron microscopy
RTCA: Real-time cell analyzer
