# Supplementary material for: IL-17F depletion accelerates chitosan conduit guided peripheral nerve regeneration
Source: Acta Neuropathol Commun. 2021 Jul 17;9:125. doi: 10.1186/s40478-021-01227-1 (PMC8285852; doi:10.1186/s40478-021-01227-1)
Supplement: Supplementary file 1 — Additional file 1. Table S1; The primer sequences for qPCR amplification. [file 40478_2021_1227_MOESM1_ESM.docx]

Table S1. The primer sequences for qPCR amplification.

| Gene | Forward primer | Reverse primer |
| --- | --- | --- |
| *Il17f* | 5’-GGGATTACAACATCACTC-3’ | 5’-AACCTGAAGGAATTAGAAC-3’ |
| *Mbp* | 5’-TTCAAGAACATTGTGACACCTC-3’ | 5’-GCCTCCGTAGCCAAATCC-3’ |
| *S100b* | 5’-TCACTTCCTGGAGGAAATCAAGG-3’ | 5’-ACACTCCCCATCCCCATCTT-3’ |
| *Nrp1* | 5’-ACCATCCAATCAGAGTTCC-3’ | 5’-AGTTGCCATCTCCTGTATG-3’ |
| *Nos2* | 5’-CGGCAAACATGACTTCAGGC-3’ | 5’-TGCACAACTGGGTGAACTCC-3’ |
| *Arg1* | 5’-TACAAGACAGGGCTCCTTTCAG-3’ | 5’-CCGTTGAGTTCCGAAGCAAG-3’ |
| *Il-1b* | 5’-CTTTGAAGAAGAGCCCATCC-3’ | 5’-CACTTGTTGGTTGATATTCTGTC-3’ |
| *Il-6* | 5’-GCTGGTGACAACCACGGCC-3’ | 5’-TCTGCAAGTGCATCATCGTTGTTCA-3’ |
| *Il-10* | 5’-GAGCTGCGGACTGCCTTC-3’, | 5’-GCAACCCAAGTAACCCTTAAAGTC-3’ |
| *Tnf* | 5’-CCTCACACTCAGATCATC-3’ | 5’-AACCTGGGAGTAGACAAG-3’ |
| *Iba-1* | 5’-ATCTGCCATCTTGAGAATGATTC-3’ | 5’-TGTGACATCCACCTCCAATC-3’ |
| *Cxcl5* | 5’-GGTCCACAGTGCCCTACG-3’ | 5’-GCGAGTGCATTCCGCTTA-3’ |
| *CD206* | 5’-CTCTGTTCAGCTATTGGACGC-3’ | 5’-CGGAATTTCTGGGATTCAGCTTC-3’ |
| *Ym1* | 5’-TTTGGACCTGCCCCGTTCAG-3’ | 5’-CTCCACAGATTCTTCCTCAAAAGC-3’ |
| *β-actin* | 5’-TGAAGATCAAGATCATTGCTCCTC-3’ | 5’- CCTGCTTGCTGATCCACATC-3’ |
| *Rn18s* | 5’-GGACAGGATTGACAGATTGATAGC-3’ | 5’-TGCCAGAGTCTCGTTCGTTATC-3’ |
